# Preparation of mono-dispersed, high energy release, core/shell structure Al nanopowders and their application in HTPB propellant as combustion enhancers

**DOI:** 10.1038/s41598-017-05599-0

**Published:** 2017-07-12

**Authors:** Fengyi Wang, Zhiguo Wu, Xushui Shangguan, Yunqiang Sun, Juanjuan Feng, Zhongyou Li, Luyang Chen, Shiyong Zuo, Renfu Zhuo, Pengxun Yan

**Affiliations:** 1 Institute for Plasma and Metal Materials, School of Physical Science and Technology, Lanzhou University, Lanzhou, 730000 China; 2 Hubei Insititute of Aerospace Chemical Technology, Xiangyang, 441000 China; 3 Institute of Nanomaterials Application Technology, Gansu Academy of Science, Lanzhou, 730000 China

## Abstract

Mono-dispersed, spherical and core/shell structure aluminum nanopowders (ANPs) were produced massively by high energy ion beam evaporation (HEIBE). And the number weighted average particle size of the ANPs is 98.9 nm, with an alumina shell (3–5 nm). Benefiting from the passivation treatment, the friction, impact and electrostatic spark sensitivity of the ANPs are almost equivalent to those of aluminum micro powders. The result of TG-DSC indicates the active aluminum content of ANPs is 87.14%, the enthalpy release value is 20.37 kJ/g, the specific heat release *S*_1_/*Δm*_1_* (392–611 °C) which determined the ability of energy release is 19.95 kJ/g. And the value of *S*_1_/*Δm*_1_* is the highest compared with ANPs produced by other physical methods. Besides, the ANPs perfectly compatible with hydroxyl-terminated polybutadiene (HTPB), 3 wt. % of ANPs were used in HTPB propellant replaced micron aluminum powders, and improved the burning rate in the 3–12 MPa pressure range and reduced the pressure exponential by more than 31% in the 3–16 MPa pressure range. The production technology of ANPs with excellent properties will greatly promote the application of ANPs in the field of energetic materials such as propellant, explosive and pyrotechnics.

## Introduction

The large specific surface area, high density, low consumption of oxygen, high volumetric heat of combustion and high reactivity made ANPs can be broadly used in propellants^1, 2^. Great attentions had been paid to aluminum nanoparticles because of their superior performances in burning and energy release, which is expected to solve the problems of aluminum micro-particles, existed in propellants^3–5^. The burning rate of the solid rocket propellants is one of the most important factors that determine the performance of rocket^6^. The typical diameter of aluminum particles used in propellants is in the order of ~30 μm^7^. The burning rate of propellants can be increased by employing aluminum powders with higher specific surface area^8, 9^. Replacement of micro-aluminum powders by ANPs will increase the propellant burning rate by ~100% and always show low pressure-exponents in 1–12 MPa pressure range^10^. Besides, the burning rate of the solid propellants increases depending on the percentage of high-energy matters, ANPs, in the propellant content^6^.

ANPs have small size and surface effects, and their surface atoms are not matched, which leads to the particles are in highly active state^2^. The high reactivity of ANPs have also caused aging problems, particularly in an environment of high relative humidity^11^. Another problem of using ANPs as additives in propellants is the original agglomeration, leading to heterogeneity of the mixtures and to coalescence of agglomerates in the heat penetration zone during combustion^12^. The near-surface combustion of ANPs controls the propellant burning rate, the high agglomeration level of ANPs points to low exponent of burning rate^10^. In order for any energetic material to have application, it must be sensitive enough to various stimuli to combust/explode under desired circumstances, and to not be ignited during handling^13^. Hence, the ANPs used for propellant needs to be treated by passivation and has good dispersion.

ANPs can be prepared using a variety of techniques, including electro-exploded wire (EEW)^14, 15^, plasma synthesized process^16, 17^, sol–gel^18^, induction heating evaporation (IHE), laser-induction complex heating evaporation (LCHE)^19^. Almost all ANPs used in propellant were prepared by physical method. In the preparation using physical methods, the aluminum nanoparticles are first collected, and then was passivated via slow oxidation or using various organic substances^20^. Characterization of ANPs includes the particle diameters, dispersion and morphology, structure, oxide layer thicknesses, thermal behavior. Among them, thermal behavior can be applied in evaluation of the reactivity of ANPs, which is the main characteristic for the application in propellants^21^.

The reactivity of ANPs, which characterizes their behavior in oxidized media was determined by four parameters which can be directly obtained from DTA (DSC) and TG curves^12, 22–24^:

1. The temperature for the onset of intensive oxidation (*T*_on_, °C),
2. The maximum rate of oxidation (*V*_ox_, mg/min),
3. The degree of conversion (degree of oxidation) of Al in a certain range of temperatures (*α*, %),
4. The ratio of the oxidation thermal effect (S/Δm*, kJ/g).

This paper developed a new technology for producing ANPs and the properties of the ANPs in HTPB propellant were studied. By high energy ion beam evaporation we produced mono-dispersed, spherical, core/shell structure ANPs. The passivation treatment of the ANPs is very effective. Although ANPs have been produced by several physical methods, the mono-dispersed ANPs were produced for the first time. The reactivity of ANPs were obtained from DSC-TG-DTG curves, the *T*_on_ of the ANPs is lower than most of the other ANPs, the specific heat release of the ANPs both in oxygen and air prepared by our method are higher than aluminum nanopowders manufactured by other physical method. The application of the ANPs in HTPB propellant increased the burning rate and significantly reduced the pressure exponent of the propellant.

## Results

### Preparation of ANPs

ANPs had been produced massively at a production rate of >200 g/h by high energy ion beam evaporation. A few nanometer thickness of dense oxide layer was formed on the surface of the particles by slowly passing through the oxygen into the vacuum chamber.

### Morphology and structure analysis

**Figure 1:**
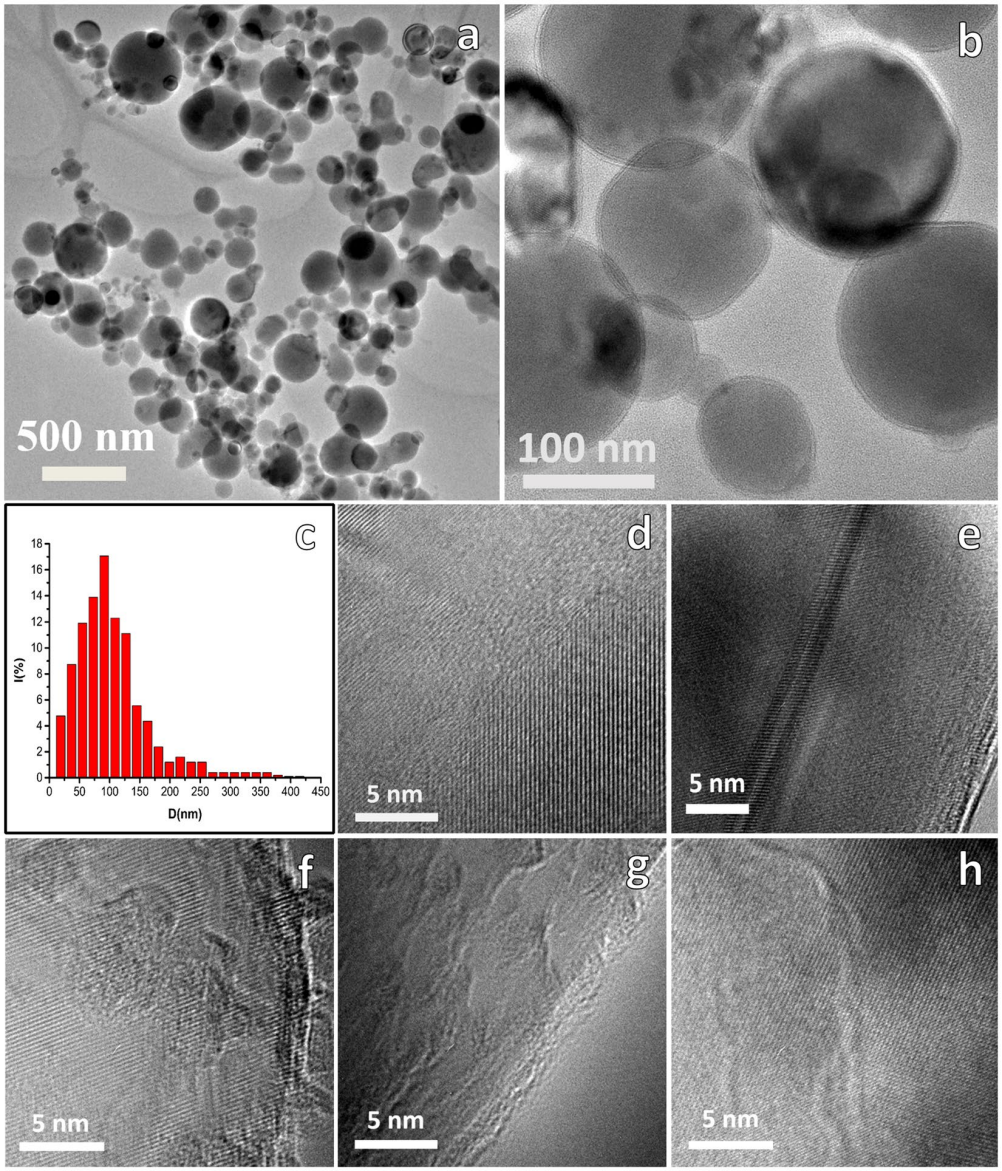
(**a**,**b**) TEM image of the sample. (**c**) Histograms showing size distribution obtained from the area of image (**a**). (**d**,**e**,**f**,**g**,**h**) HRTEM image of the sample that showed the lattice distortion and the structural characteristics of the interface.

The characteristics of the ANPs are given in Table 1. The morphology, particle size distribution and passivation layer of the sample are shown in Fig. 1. It can be seen from Fig. 1a that the particles are mostly spherical in shape and the particle size distribution (Fig. 1c) obtained from Fig. 1a showing that most particles distribute in 20 to 200 nm, 48.3% of the particles are less than 100 nm, and the number weighted average particle size (*D*_n_) is about 98.9 nm. As can be seen from Fig. 1b and Supporting Information Fig. S1, the ANPs are core-shell structure, with a thin shell (3–5 nm), and the shell is amorphous. This can be proved by the XRD and EDS results. The lattice distortion and the structural characteristics of the interface are shown in Fig. 1d–h. As shown in Fig. 1d of high resolution, the particles are polycrystalline crystals composed of different oriented single crystals.

**Table 1:** The characteristics of the ANPs.

Sample	Structure	Shape	Dispersion	*S*_sp_, m^2^/g	*D*_n_, nm	*D*_s_, nm	*D*_g_, nm
ANPs	Core/shell	Sphere	Mono-dispersed	20.92	98.9	106.2	36.1

**Figure 2:**
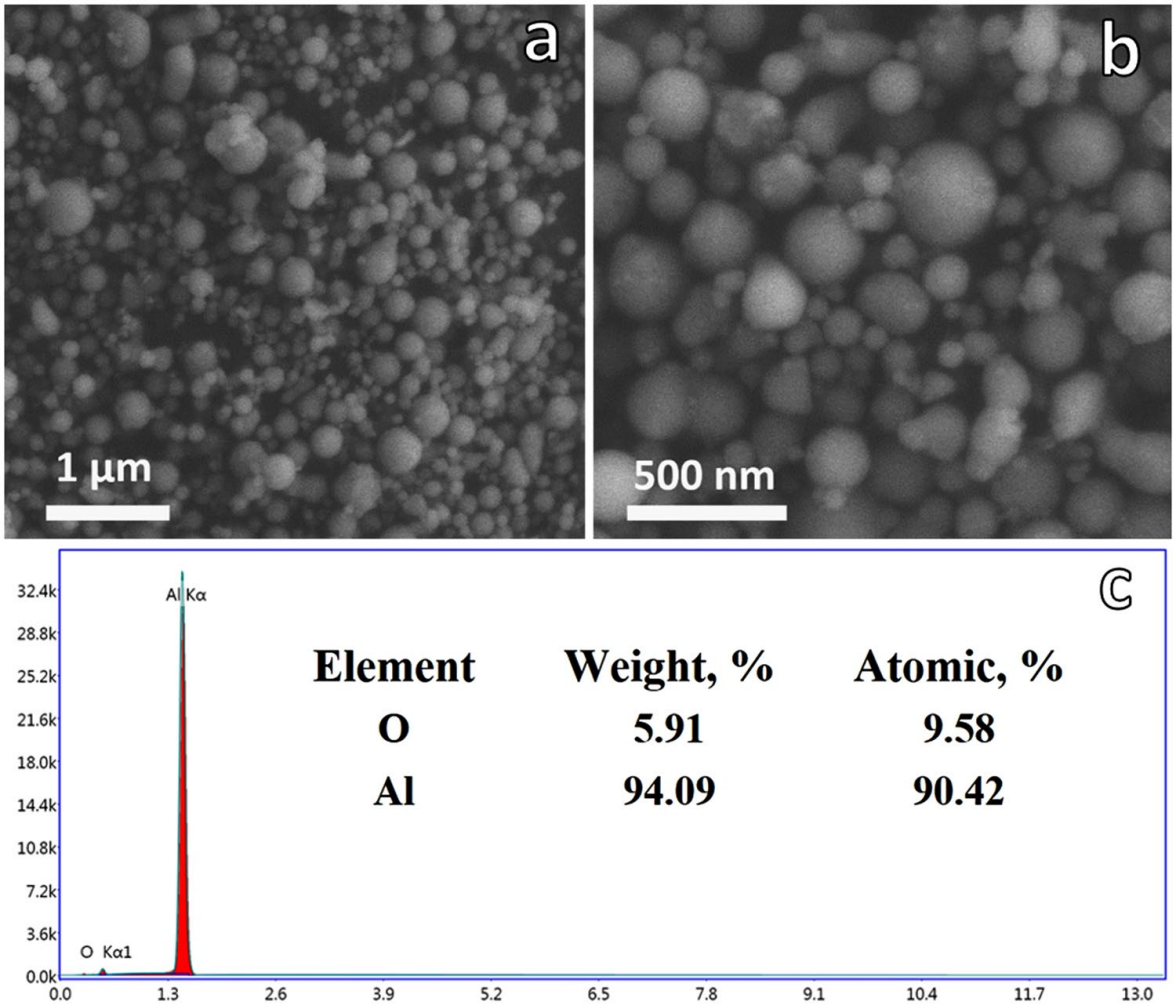
(**a**,**b**) SEM graph of the sample. (**c**) Energy-dispersive spectroscopic (EDS) result of the sample.

Figure 2 is SEM images of the sample, and it was observed that the particles were spherical and mono-dispersed. The EDS result (Fig. 2c) implies 87.44% of Al and 12.56% of Al_2_O_3_, assuming the ANPs only contain Al and Al_2_O_3_ compounds.

**Figure 3:**
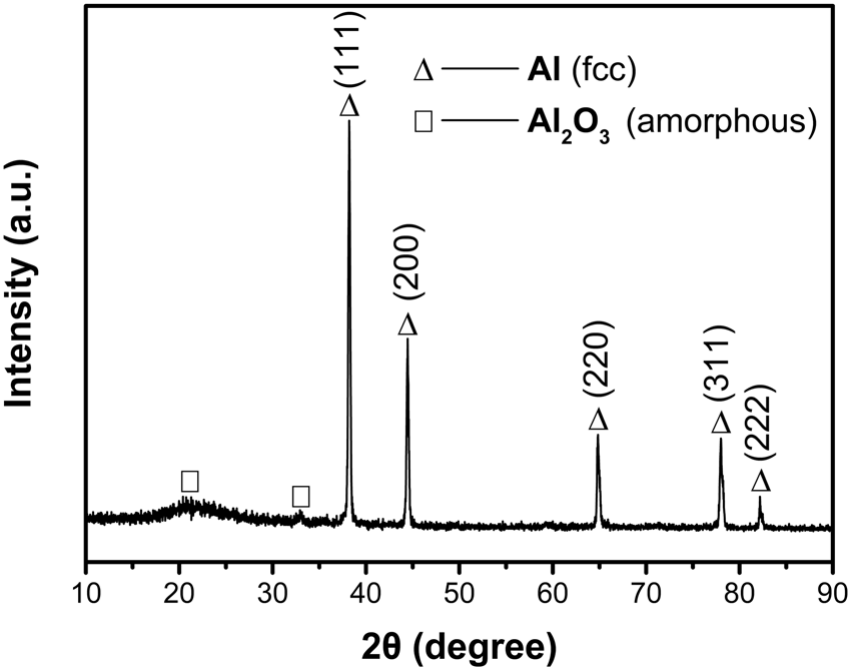
XRD diffraction spectra of the sample.

X-ray diffraction (XRD) pattern of the sample is shown in Fig. 3. The strong diffraction peaks appears at 2*θ* = 38.26°, 44.52°, 64.90°, 78.06° and 82.24°. The peak positions are in good agreement with the JCPDS file (no. 04–0787), which belongs to the face-centered cubic (f. c. c) structure of aluminum (111), (200), (220), (311) and (222). The absence of any crystalline alumina peaks indicates that the alumina shell is mainly amorphous as shown in Supporting Information Fig. S1. Two weak wide peaks appear at 15–35° which correspond to the amorphous Al_2_O_3_. The result means the sample mainly contains metallic Al and a small quantity of Al_2_O_3_, which is in good agreement with the EDS result.

The *D*_g_ which can be approximately obtained by Scherrer equation (Eq. (1)) is also estimated by XRD^25, 26^:

1 $${D}_{g}=K\lambda /(B\cdot \cos \,\theta )$$

where, Scherrer constant K = 0.89, the λ = 0.154187 nm, *B* is half-height of the diffraction peak, *θ* is the Bragg diffraction angle. The average grain size for the ANPs was obtained from two peaks (2*θ* = 38.261°, 44.520°).

The average particle size of the spherical particles is also estimated as follows^24^:

2 $${D}_{s}=6000/(\rho \cdot {S}_{sp})$$

where, ρ_Al_ – density of aluminum (2.7 g/cm^3^); *S*_sp_ – BET surface area of the sample, m^2^/g. The *S*_sp_ of the sample is 20.92 m^2^/g, *D*_*s*_ = 106.2 nm.

The average particle size D_n_ and D_s_ is approximate equal, and they are almost three times of D_g_, which proved that the particles are polycrystal, and the big nanocrystalline particles consist of many small randomly oriented grains, which has been verified in Fig. 1d.

### Thermal analysis of the ANPs

**Figure 4:**
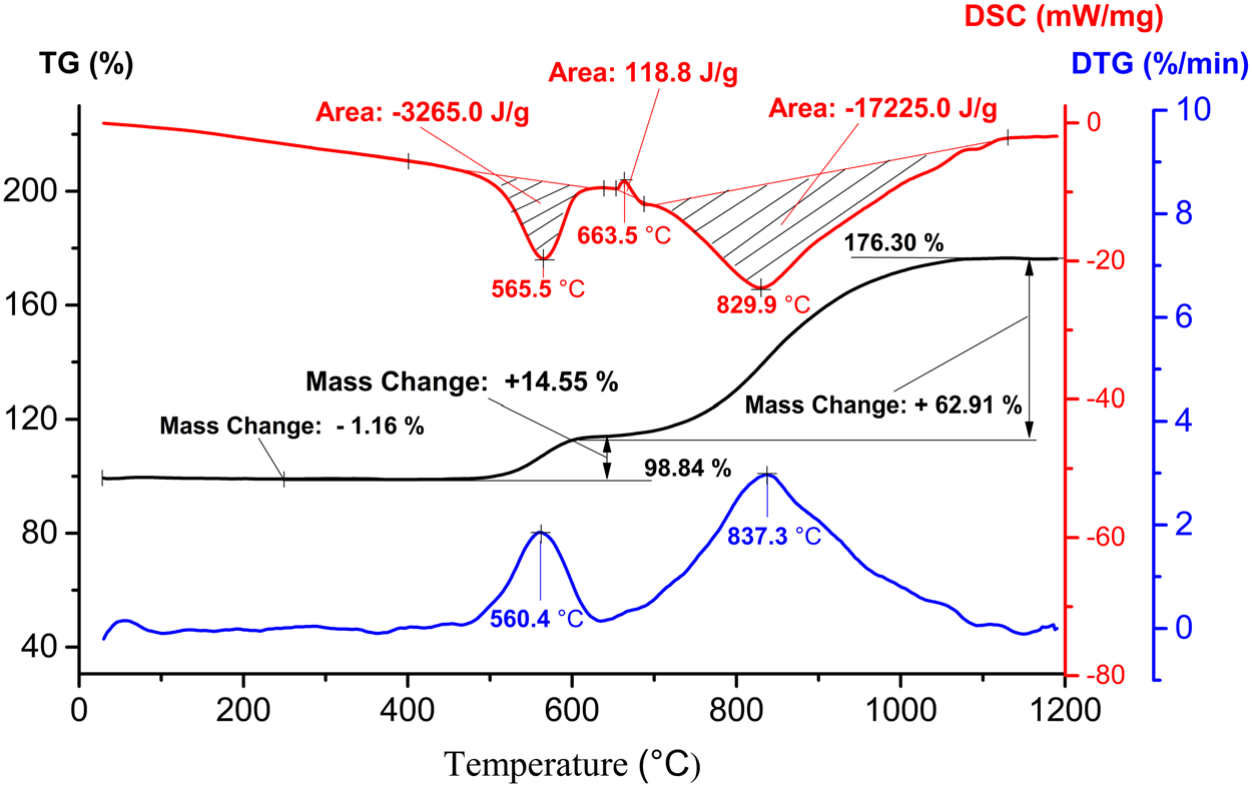
DSC-TG-DTG results for the sample heated in oxygen at 10 °C/min.

The TG-DTA-DSC results for ANPs in oxygen are shown in Fig. 4. Reactivity parameters of the ANPs in oxygen are given in Table 2. The TG-DTA-DSC results for ANPs in air are shown in Supporting Information Fig. S2.

**Table 2:** Reactivity Parameters from DSC-TG-DTG Analyses of ANPs.

Sample	*T*_on_, °C	*Δm*, %	*ΔH*, kJ/g	S/Δm*, kJ/g	*V*_ox_, mg/min	*α*, %	*ΔH*_r_, kJ/g
ANPs (1.44 mg)	509	14.55 (392–611 °C)	3.265	19.95	0.0267	87.14	20.37
		60.38 (700–1100 °C)	17.225	25.36	0.0428		

From Fig. 4 and Supporting Information Fig. S3, it was found that the weight lossed 1.16% and then begin to oxide, the oxidation of ANPs occurs in four macroscopic stages: the first stage begins at 392 °C as shown in Supporting Information Fig. S3, the percent weight gain was 1.39%; the second stage, it is the intense stage, initiates at 509 °C, which was the temperature for the onset of intensive oxidation (*T*_on_) determined from DSC curves by the widely used tangent-curves method as shown in Supporting Information Fig. S4, in this stage the weight gained 13.25%; Then a distinct decreasing trend is observed with the exception of the ANPs oxidized at 611 °C, up to the first pseudoplateau, the percent weight gain was 2.53%; and the fourth stage, it is the second intense stage, oxidation of residual aluminum, begins at about 700 °C and continues up to full oxidation of the aluminum at 1100 °C. During the first two stages, the percent mass gain *Δm*_1_(%) was 14.55%, and the enthalpy change *ΔH*_1_ determined by the exothermic peak area was calculated by the DSC thermal analysis system and the result is 3.265 kJ/g, the maximum rate of oxidation *V*_ox1_ determined by DTG curve is 0.0267 mg/min. At the second intense stage, *Δm*_2_(%) was 60.38%, *ΔH*_2_ = 17.225 kJ/g, *V*_ox2_ = 0.0428 mg/min.

The mass gain in the TGA is attributed to oxidation of active aluminum, as shown by the following reaction^22^:

3 $$4{\rm{Al}}+3{{\rm{O}}}_{2}\to 2{{\rm{Al}}}_{{\rm{2}}}{{\rm{O}}}_{3}$$

The active aluminum content can be calculated using the following equation (Eq. (4)):

4 $$\alpha ( \% )=108/96\cdot {\rm{\Delta }}m( \% )$$

where *Δm*(%) is the percent mass gain, which determined from TG curves is 77.46% as shown in Supporting Information Fig. S3. The *α*(%) of the ANPs is shown in Table 2.

A small endothermic peak (655–687 °C) is observed in DSC curve, it was caused by melting of the sample^27^. The melting point of ANPs which determined from DSC curves by tangent-curves method was 656 °C as presented in Supporting Information Fig. S4 (4 °C below the bulk aluminum melting point 660 °C^25, 28^).

The enthalpy release of the ANPs during the oxidation progress was

5 $${\Delta }{H}_{{\rm{r}}}=\Delta {H}_{1}+{\Delta }{H}_{2}-{\Delta }{H}_{{\rm{f}}}=20.37\,{\rm{k}}{\rm{J}}/{\rm{g}}.$$

The specific heat release is given by the following formula (Eq. (6)), which is a parameter to determine the ability of energy release^19^:

6 $${S}_{{\rm{i}}}/{\Delta }{m}_{{\rm{i}}}^{\ast }={\Delta }{H}_{{\rm{i}}}\cdot {m}_{0}/(1.125{\Delta }{m}_{{\rm{i}}}\cdot {m}_{0})={\Delta }{H}_{{\rm{i}}}/(1.125{\Delta }{m}_{{\rm{i}}})$$

where *S*_i_ is the heat release of the sample within a certain temperature range, *m*_0_ is the initial mass of the tested sample, *ΔH*_i_ is the enthalpy change. The specific heat release of the ANPs can be seen in Table 2.

### Application in HTPB propellant

- Sensitivity performance of ANPs. The friction, impact and electrostatic discharge (ESD) sensitivity of ANPs and micro aluminum powders are shown in Table 3. It can be seen that the friction, impact and electrostatic discharge sensitivity of the ANPs and micron aluminum powders are basically the same.

**Table 3:** Compared the sensitivity of ANPs and aluminum micron powders.

Type		Test result		Test condition
		ANPs	AMPs (30.96 μm)	
ESD	*V*50, V	1417.0	1533.3	Temperature 25 °C, Relative Humidity 50%, Capacitance 3 × 3900 P_F_, Stitch Length 0.5 mm
	*E*50, mJ	11.75	13.75	
Friction sensitivity		0%	0%	Temperature 23 °C, Relative Humidity 70%, Testing Angle 90°, Test Pressure 4.0 MPa
Impact sensitivity		0%	0%	Temperature 23 °C, Relative Humidity 70%, Drop Weight 98.0 N, Drop height 50 cm, Impact Energy 49 J
- Compatibility of the ANPs and HTPB propellant. The result shown in Table 4 proves the compatibility of ANPs and HTPB propellant is very well.

**Table 4:** Compatibility of ANPs and HTPB propellant.

Sample	Initial mass, g	Mass after 14 days, g	Isothermal weight loss rate, %
Conventional HTPB propellant	9.887	9.879	0.081
Adding 3 wt.% ANPs HTPB propellant	10.499	10.493	0.057
- Effect of ANPs on burning rate of HTPB propellant. The burning rate of the HTPB propellant consisting 3 wt. % ANPs and the conventional HTPB propellant under low and high pressure are shown in Supporting Information Table S1, Table S2 and Fig. 5. The results show that the addition of 3 wt. % ANPs can increase the burning rate of HTPB propellant at low pressure (3.0 MPa, 5.0 MPa, 7.0 MPa, 9.0 MPa), and reduce the pressure exponential by 31.82%. At high pressure (10.0 MPa, 12.5 MPa, 14.0 MPa, 16.0 MPa), the burning rate under the pressure of 12.5 MPa was increased, and the pressure exponential of whole high pressure section from 0.86 to 0.56 was reduced (percentage reduced is 34.88%).

**Figure 5:**
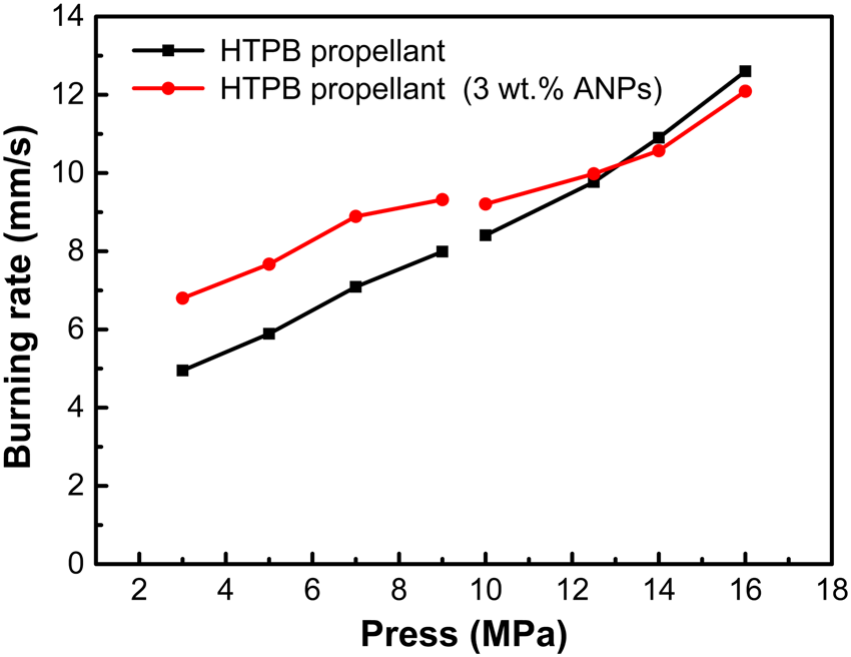
Effect of ANPs on burning rate of HTPB propellant.

## Discussion

Because ion beam can continuously provide very high energy, high surface powder density, raw materials rapidly evaporation, ANPs were massively produced, which can realize the industrial production of aluminum nanopowders at a production rate of >200 g/h. The collecting wall was kept under 25 °C. The temperature gradient between vapor generating zone and collecting wall was huge, aluminum vapor was subjected to rapid cooling via cooling zone. By the rapid homogeneous nucleation and quenching, the mono-dispersed nanometer aluminum powders was produced. In order to prevent the agglomeration of ANPs particles, we performed a slow passivation process. This passivation treatment is very effective, resulting the particles keep mono-dispersion and the friction, impact and ESD sensitivity of the ANPs almost equivalent to the micro aluminum powders. In order to apply ANPs in propellant, it must not be ignited during handling. Friction, impact and ESD sensitivity are important aspects^13^. The micron powder in the propellant has been applied maturely, so the ANPs can be safely used in the propellant and other energetic materials.

The HRTEM and XRD results show that the particles consist of many small randomly oriented grains. The microstructure of the particles can be viewed as a two-phase system: highly-constrained metastable crystalline in the grain interiors connected by the serious-constrained amorphous grain boundaries (GBs), glue-like phase^25^. There is a large number of grain boundary inside the particles, which have a certain effect on the thermal properties of ANPs.

In oxygen atmosphere, from 30 °C to 392 °C the weight loss of the sample is 1.16% due to the desorption of the water vapor, CO_2_ and other gas adsorbed on the surface of the particles under heating condition^29, 30^. During the first stage (392–509 °C), the weight gain 1.39%. The possible reason was that some thicker amorphous alumina regions reached their critical nucleus for crystallization owing to the slight oxidation^27^. In second stage (509–611 °C), a core−shell oxidation takes place through the oxide shell by inward diffusion of oxygen or outward diffusion of aluminum^27^, oxygen diffusion may predominate once the alumina shell is crystallized^31, 32^, occurs for 13.25% mass gain. Then a slight decreasing trend of the mass gain is observed at about 611 °C, that is, up to the first plateau in TGA curve. The oxygen needed to pass through the oxide layer formed on previous stage to reach the reaction layer of aluminum core and reacted with the aluminum during this stage^33^. Besides, the density of the alumina shell becomes larger through the crystallization process, the alumina layer is denser^34^, the diffusion of oxygen is more difficult^32^, resulting the appearing of the plateau, in this stage the weight gain 2.53%. The fourth stage (700–1100 °C), the weight gain 60.38%, above 700 °C the oxidation rate restarts, the aluminum core melt and its volume expand, predominating by the outward diffusion of aluminum ions. The aluminum diffuses through the oxide shell and the reaction interface is located at the external surface of the particles^27^. Up to 1100 °C, there is no aluminum, which has been confirmed by the XRD result as shown in Supporting Information Fig. S5.

The fusion enthalpy (*ΔH*_f_) of the ANPs was only 30% of the bulk aluminum (396 J/g)^35^ since at 656 °C, some of aluminum had been oxidized, resulting less available aluminum (108/96·61.99% = 69.74%) for melting and hence the decreased enthalpy of melting. Besides, the large interfacial energy resulted from the large volume fraction of grain boundaries led to the less of enthalpy of melting, using the Gibbs–Thomson equation Eq. (7)^35, 36^:

7 $${\Delta }{H}_{{\rm{f}}}(r)={\Delta }{H}_{{\rm{f}}}(\infty )-2{\sigma }_{{\rm{sl}}}/({\rho }_{s}\cdot r)$$

where *σ*_sl_ is solid–liquid interfacial energy and *ρ*_s_ is the solid phase density, *ΔH*_f_ (*r*) is the heat of fusion for a particle with radius *r*, *ΔH*_f_ (*∞*) is the heat of fusion for bulk aluminum.

During 700–1100 °C, the ability of energy release of ANPs is 27% higher than the value at 392–611 °C. In most reports, the DSC test temperature is lower than 800 °C, and the specific heat release below 650 °C was used to determine the ability of energy release of ANPs. The *S*_1_/*Δm*_1_* of ANPs is higher than that of the other ANPs, and the percentage of *S*_1_/*Δm*_1_* of our ANPs higher than other ANPs has been shown in Table 5. The ability of energy release of aluminum nanopowders both in oxygen and air prepared by our method are higher than aluminum nanopowders manufactured by other physical method below 650 °C as shwon in Table 5. During the production process ion beam provided very high energy, part of the heat energy stored into the particles. The particles can store a largr volume of excess enthalpy that can even exceed its fusion enthalpy, and the stored enthalpy is regarded to be closely related to the lattice distortion and the structural characteristics of the interface^25^ (as shown in Fig. 1d–h).

**Table 5:** Compared the ability of energy release below 650 °C of ANPs manufactured by different methods.

Method	*D*, nm	Measurement atmosphere	*T*_*on*_,°C	*ΔH*, kJ/g	*Δm*_1_,%	*S*_1_ */Δm*_1_*,kJ/g	*S*_1_ */Δm*_1_* of our sample in O_2_ higher than other ANPs, %	Reference
HEIBE	98.9	O_2_	509	3.265	14.55	19.95	—	our sample
		Air	516	3.082	14.15	19.36	2.9	
LCHE	20.0–50.0	O_2_	529	3.548	16.80	18.77	6.1	19, 21
Laser method	50.0	O_2_	556	4.219	22.00	17.05	17.0	3, 21
Plasma technology	90.0	Air	430	5.500	32.00	15.28	30.6	38
Induction method	50.0	O_2_	553	3.584	22.80	13.97	42.8	3
EEW(Alex)	183.0	Air	460	3.900	25.00	13.87	43.8	11, 39
IHE	20.0–50.0	O_2_	530	1.180	19.80	5.30	276.4	19

The temperature for the onset of intensive oxidation (*T*_on_, °C) as shown in Table 2, which is 147 °C lower than the melting point of the ANPs and the *T*_on_ of the ANPs made by HEIBE was lower than that of most ANPs made by other methods. As reported by other researchers^23^, the onset temperature for oxidation of aluminum nanoparticles decreases with the decreasing of particle size. We think that the low *T*_on_ of ANPs was due to the distribution of the particles from 20 to 200 nm, 48.3% of the particles are less than 100 nm, the polycrystalline particles consist of many small randomly oriented grains with the volume weighted mean grain size is 36.1 nm, consequently resulting in large volume fraction of grain boundaries. The interfacial energy is released when heating the sample, which helps to reduce the *T*_on_ and improve the *S*_1_ */Δm*_1_*.

For the safty consideration in the propellant application of the ANPs, its friction, impact and ESD sensitivity factor should achieve the application requirement. The sensitivity of ANPs and micro powders are shown in Table 3. It can be seen that the friction, impact and electrostatic discharge sensitivity of ANPs and micron powders are basically the same. The passivation process is proved to be effective. The micron powder in the propellant has been applied maturely, and the ANPs can be safely used in propellants.

In this paper we use the sedimentation of the particles in the HTPB to evaluate the compatibility of the ANPs and HTPB. As the ANPs is mono-dispersion, it has a high specific surface areas, the particles are able to fully contact with the HTPB, decreasing the settlement of the particles and has a good compatibility with the HTPB.

Because of its excellent ability of energy release, low temperature for the onset of intensive oxidation, high active aluminum content of 87.14%, high enthalpy release value of 20.37 kJ/g, the ANPs is well apply to propellant, explosive, pyrotechnic formulations and other energy materials.

With a large specific surface area of the ANPs, the thickness of the aluminum particle is in the nanoscale, the heat release of aluminum combustion to occur close to the propellant surface, causing the increase of burning rate^8^. 3% of ANPs mixing with micro aluminum powder used in HTPB propellant can increase the burning rate in the 3–12 MPa pressure range and the percentage increase in burn rates reduced with the increase of pressure. On the other hand, the pressure exponential reduced by more than 31% in the 3–16 MPa pressure range and reduced more in high pressure range. In particular, small values of the pressure exponent, *n*, are aimed for, in order to avoid transition to unstable burning at high pressures^37^. Hence, the addition of ANPs significantly improved the stability of HTPB propellant, especially at high pressure condition.

In the future work, more ion beams can be added to supply more power for the evaporation system to increase the yield of ANPs. Using this technology, many metals and alloy nanopowders could be industrially produced. A lot of researches on the ANPs in HTPB propellant could be tried.

## Methods

### Preparation of ANPs

The ANPs were produced by high energy ion beam evaporation (HEIBE). The equipment consists of the power supply, cooling system, vacuum unit, graphite crucible, high energy ion beam system and powder collection system. A high energy ion beam system was equal interval installed on the upper part of the side of the cylinder vacuum chamber. The bottom diameter of the vacuum chamber was 80 cm and its height was 80 cm. Besides, the ion beam with 60° irradiated into the crucible. In the process of the production, the power supplies power of 20 kW for the evaporation system. A mechanical pump and a diffusion pump were used to evacuate the system to get a base pressure of 10^-3^ Pa. Aluminum blocks or powders (99.9% pure) in the graphite crucible were used as raw materials. The ion beam provided enough heat to ensure the stability of the production of aluminum vapor. Circulating water cooling system was used to prove the necessary cooling for the powder collection system. The collecting wall was kept at 20–25 °C. The aluminum vapor was subjected to rapid cooling via cooling zone, before they reached to the collecting wall. ANPs were formed due to the rapid homogeneous nucleation and quenching. After the nanopowders were produced, oxygen was slowly access in 5 sccm to form passivation layer. Five hours later, filled air into the collection system up to atmospheric pressure and then collected the powders after several minutes for static.

### Characterization of the ANPs

The morphology, particle size distribution and passivation layer of ANPs were estimated by transmission electron microscopy (TEM, FEI Tecnai F30). Scanning electron microscopy (SEM, Hitachi S-4800) was also used to directly estimate the morphology and dispersion property, and the chemical microanalysis was obtained by energy-dispersive spectroscopic (EDS).

The volume weighted mean grain size (*D*_g_), phases and crystal structure of the ANPs was examined by X-ray diffraction (XRD) technique using X-ray Diffractometer (D/max-2400) with a Cu Kα source (λ_Kα_ = 1.54187 Å) at a measurement angle range 2*θ* = 10–90°. The specific surface areas (*S*_sp_) of the ANPs were quantified by nitrogen adsorption−desorption isotherms analyses performed at 77.35 K using the Quantachrome NOVA Station 1 apparatus. Before adsorption measurement, the sample was evacuated at 100 °C under vacuum for 6 h. The *S*_sp_ of the sample was determined by Brunauer-Emmet-Teller (BET) method.

### Thermal analysis

NETZSCH STA 449F5 Simultaneous TG-DSC Thermal Analysis System was used for the thermal studies of ANPs in oxygen and air. Sample of 1.44 mg was placed in alumina pans and heated in oxygen (10 sccm, 99.999%) at 10 °C/min from 30 to 1200 °C. And 1.41 mg sample was heated in air at 10 °C/min from 30 to 1200 °C. DSC-TG-DTG curves were obtained.

### Application of ANPs in HTPB propellant

3 wt. % of ANPs were used in HTPB propellant to replace aluminum micro powders (AMPs, *D*_50_ = 30.96 μm). Friction, impact and electrostatic discharge sensitivity (ESD) of ANPs were determined, and compared with micro powders which the test condition is given in Table 3. The compatibility of ANPs and HTPB propellant was tested by Isothermal Thermogravimetric Method at 70 °C for 14 days. Static burning rate and exponent of the conventional HTPB propellant (18 wt. % AMPs) and the adding ANPs HTPB propellant (3 wt. % ANPs and 15 wt. % AMPs) under low pressure (3.0 MPa, 5.0 MPa, 7.0 MPa, 9.0 MPa) and high pressure (10.0 MPa, 12.5 MPa, 14.0 MPa, 16.0 MPa) were also estimated by constant pressure static burning rate tester (WAE 2000).

## Electronic supplementary material

Supporting Information
