# Supplementary material for: Preparation of mono-dispersed, high energy release, core/shell structure Al nanopowders and their application in HTPB propellant as combustion enhancers
Source: Sci Rep. 2017 Jul 12;7:5228. doi: 10.1038/s41598-017-05599-0 (PMC5507889; doi:10.1038/s41598-017-05599-0)
Supplement: Supplementary file 1 — Supporting Information [file 41598_2017_5599_MOESM1_ESM.docx]

Supporting Information

**Preparation of mono-dispersed, high energy release, core/shell structure Al nanopowders and their application in HTPB propellant as combustion enhancers**

Fengyi Wang^1^, Zhiguo Wu^1,3,*^, Xushui Shangguan^2^, Yunqiang Sun^1^, Juanjuan Feng^1^, Zhongyou Li^2^, Luyang Chen^2^, Shiyong Zuo^1^, Renfu Zhuo^1^ and Pengxun Yan^1,3^

^1^ Institute for Plasma and Metal Materials, School of Physical Science and Technology, Lanzhou University, Tianshui Road, Lanzhou, 730000, Gansu, China.

^2^ Hubei Insititute of Aerospace Chemical Technology, Chunyuan Road，Xiangyang, 441000, Hubei, China.

^3^ Institute of Nanomaterials Application Technology, Gansu Academy of Science, Dingxi Road, Lanzhou, 730000, Gansu, China.

^*^ Corresponding Authors: [zgwu@lzu.edu.cn](mailto:zgwu@lzu.edu.cn)


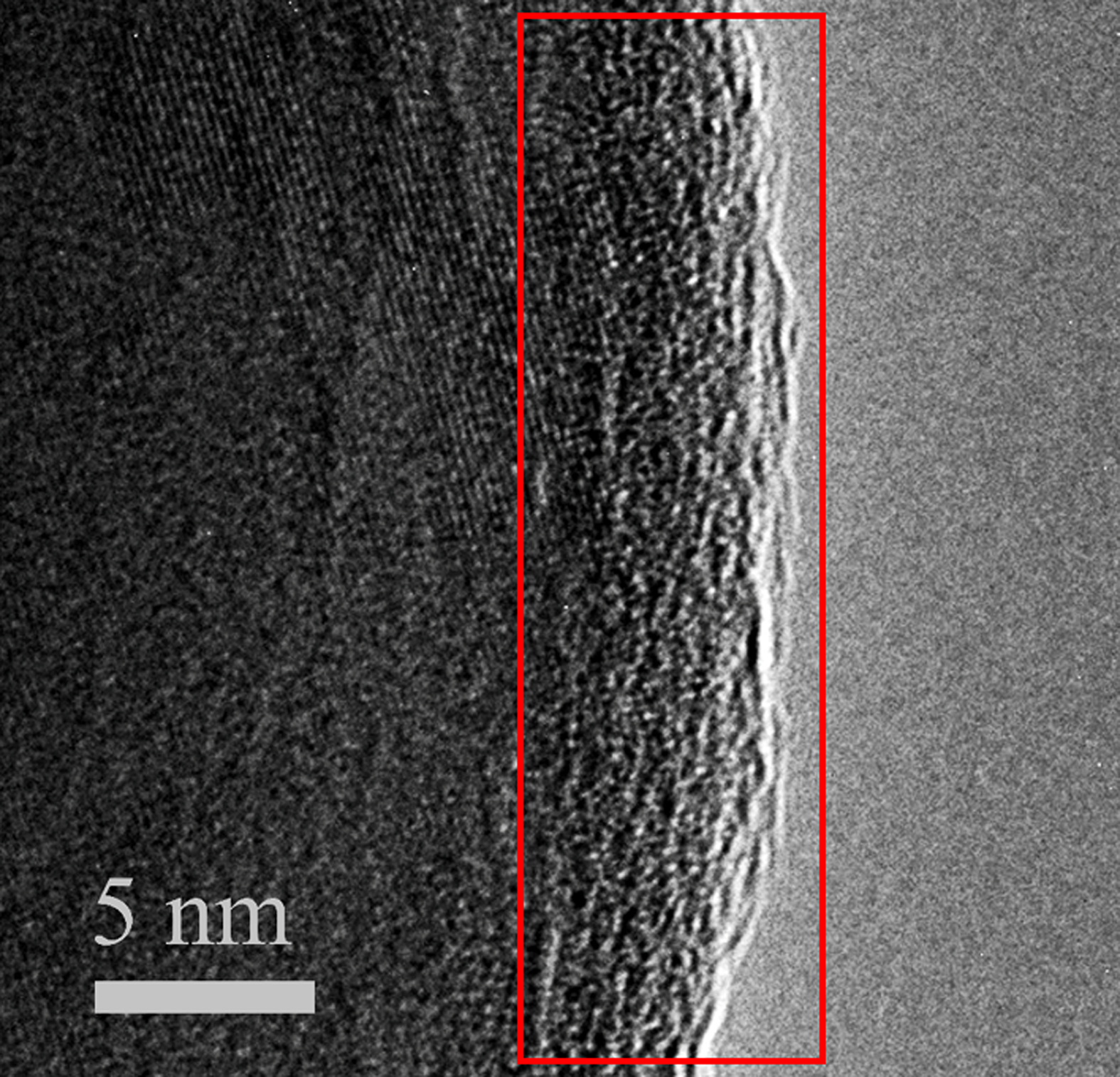


**Figure S1.** HRTEM image of the sample that showed the amorphpus alumina shell.


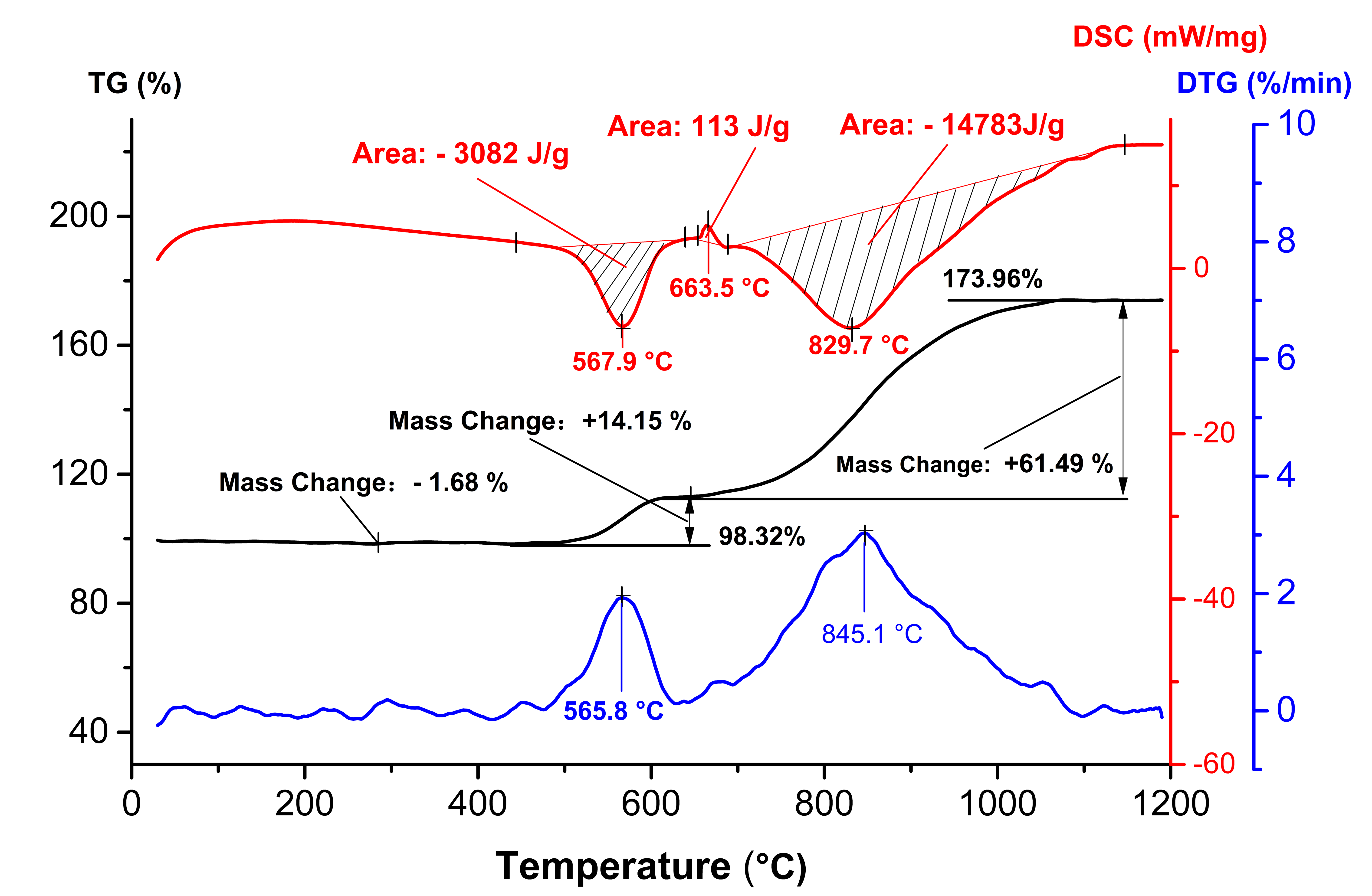


**Figure S2.** DSC-TG-DTG results for the sample heated in air at 10 °C/min.

**
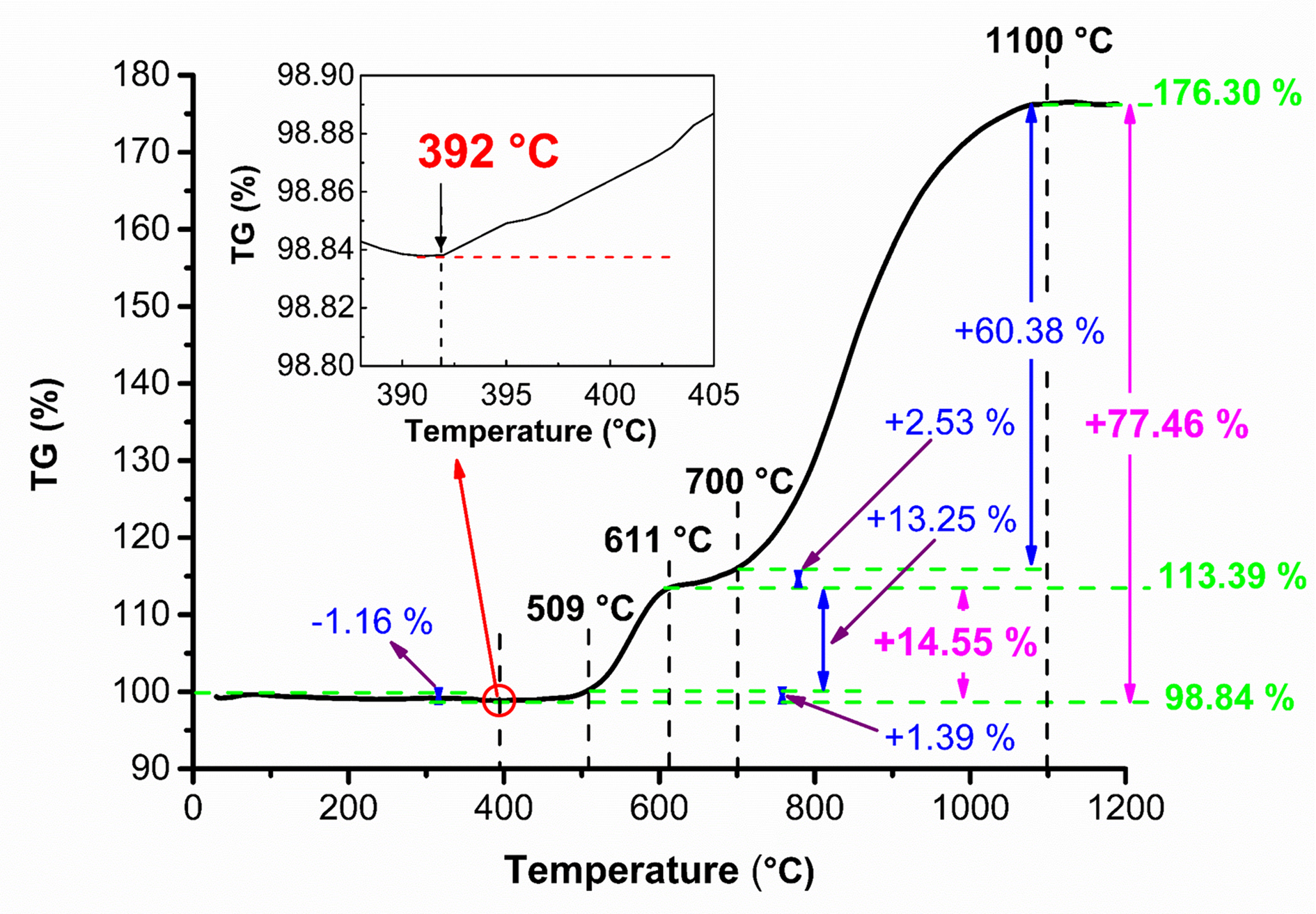
**

**Figure S3.** TG result for the sample heated in oxygen at 10 °C/min.


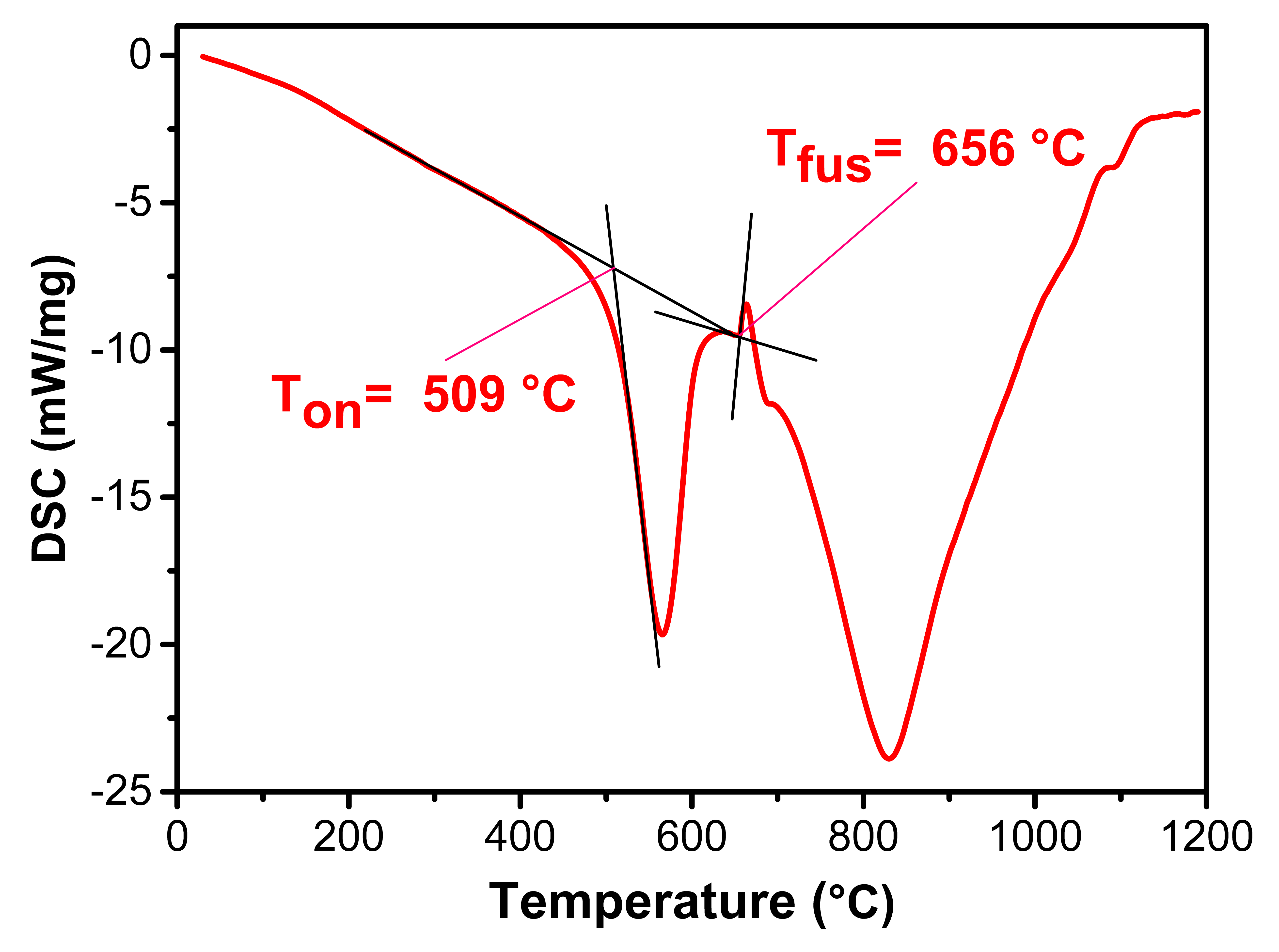


**Figure S4.** The temperature of intensive oxidation onset (*T_on_*, °C) and the melting point (*T_fus_*, °C) of the sample were determined from DSC curves by tangent-curves method.


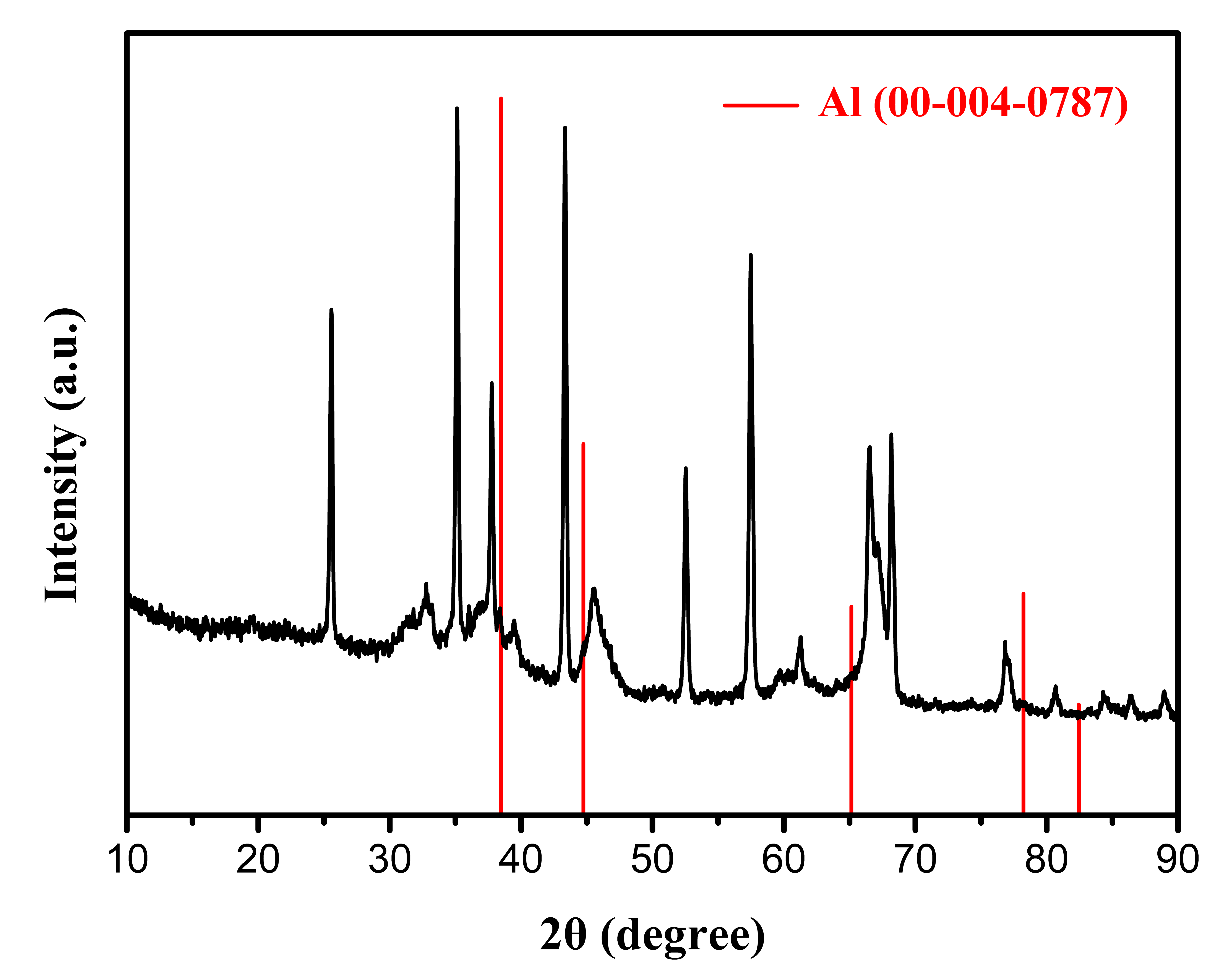


**Figure S5.** XRD diffraction spectra of the sample heated from 30 °C to 1100 °C in oxygen at 10 °C/min, showing that there is no peak corresponding to metallic Al.


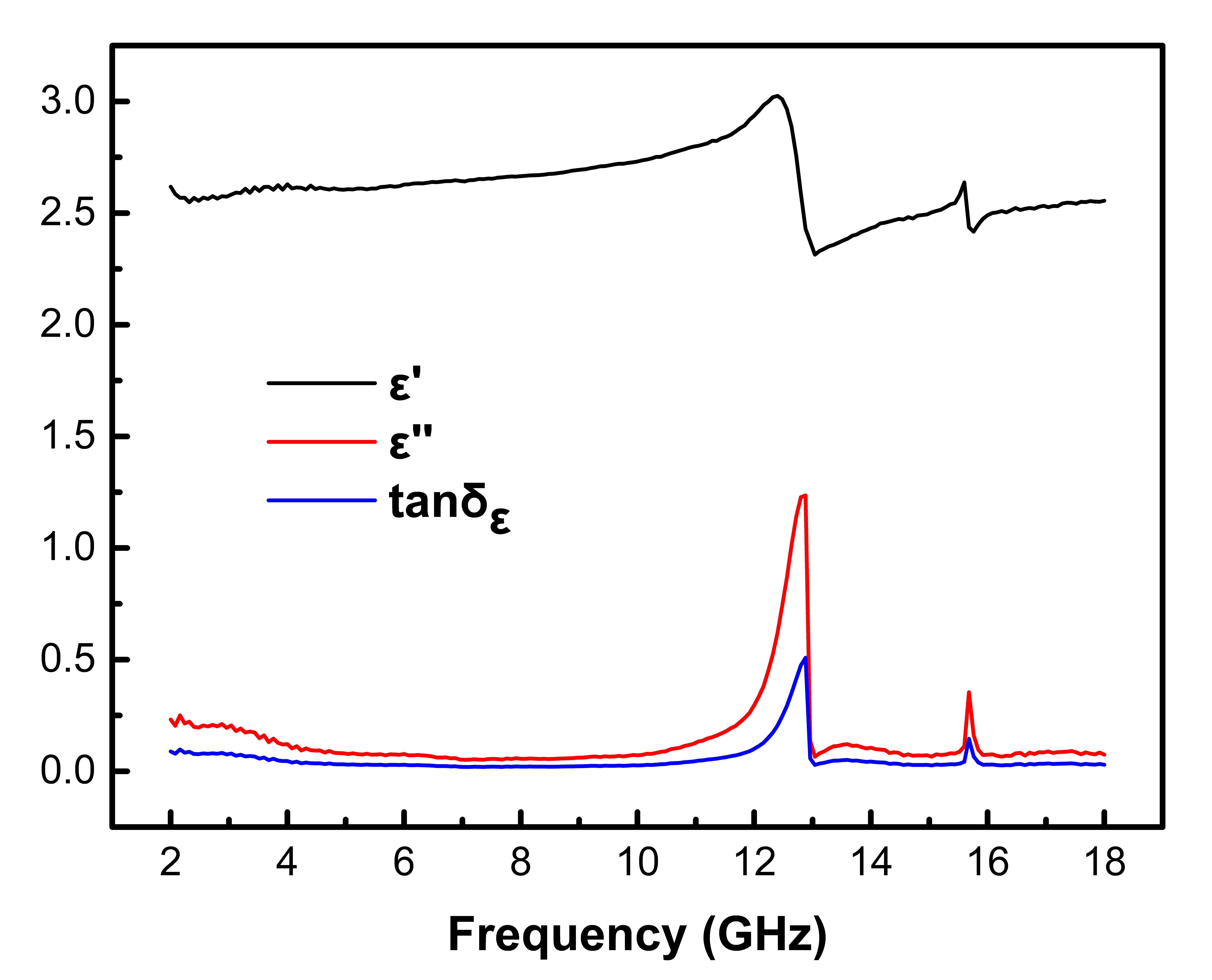


**Figure S6.** The complex dielectric constant (*ε*_r_) and dielectric loss factor (*tanδ_ε_*) of the ANPs.

|  | **Burning rate *v*, mm/s** | | | | **Exponent *n*** |
| --- | --- | --- | --- | --- | --- |
| Pressure | 3.0 MPa | 5.0 MPa | 7.0 MPa | 9.0 MPa |  |
| Conventional HTPB propellant | 4.95 | 5.89 | 7.09 | 7.99 | 0.44 |
| Adding 3 wt.% ANPs HTPB propellant | 6.80 | 7.67 | 8.89 | 9.32 | 0.30 |
| Percentage increase, % | 37.37 | 30.22 | 25.39 | 16.65 | -31.82 |

**Table S1.** Burning rate test at low pressure.

|  | **Burning rate *v*, mm/s** | | | | **Exponent *n*** |
| --- | --- | --- | --- | --- | --- |
| Pressure | 10.0 MPa | 12.5 MPa | 14.0 MPa | 16.0 MPa |  |
| Conventional HTPB propellant | 8.41 | 9.77 | 10.9 | 12.6 | 0.86 |
| Adding 3 WT.% ANPs HTPB propellant | 9.21 | 9.98 | 10.57 | 12.09 | 0.56 |
| Percentage increase, % | 9.51 | 2.15 | -3.03 | -4.05 | -34.88 |

**Table S2.** Burning rate test at high pressure.
